# The Impact of Social Support on Public Anxiety amidst the COVID-19 Pandemic in China

**DOI:** 10.3390/ijerph17239097

**Published:** 2020-12-06

**Authors:** Yibin Ao, Hao Zhu, Fanrong Meng, Yan Wang, Gui Ye, Linchuan Yang, Na Dong, Igor Martek

**Affiliations:** 1 College of Environment and Civil Engineering, Chengdu University of Technology, Chengdu 610059, China; aoyibin10@mail.cdut.edu.cn (Y.A.); zhuhao704538478@163.com (H.Z.); mengfanrong0913@163.com (F.M.); 2 Department of Engineering Management, Sichuan College of Architectural Technology, Deyang 618000, China; wangyan_hy09@sina.com; 3 School of Management Science and Real Estate, Chongqing University, Chongqing 400045, China; yegui760404@126.com; 4 School of Architecture and Design, Southwest Jiaotong University, Chengdu 611756, China; 5 College of Architecture and Environment, Sichuan University, Chengdu 610065, China; dongna@scu.edu.cn; 6 School of Architecture and Built Environment, Deakin University, Geelong, VIC 3220, Australia; igor@deakin.edu.au

**Keywords:** social support, public anxiety, State–Trait Anxiety Inventory, Social Support Rating Scale, mental health, epidemic, pandemic, COVID-19, 2019 novel coronavirus (2019-nCoV), China

## Abstract

The recent coronavirus outbreak has captured worldwide attention. This study investigated the anxiety of the Chinese public and its relationship with social support during the early stage of the COVID-19 pandemic, thereby providing empirical support for interventions on improving the public’s mental health. On the basis of an online questionnaire survey conducted on 10–18 February 2020, this study shows that 19.8%, 68.5%, and 11.1% of the respondents suffered mild anxiety, moderate anxiety, and severe anxiety, respectively. Significant differences are reported in state anxiety between people with different household incomes. There are significant differences in trait anxiety and state anxiety between different social support groups. Social support and trait anxiety are negatively correlated. Social support and state anxiety are negatively correlated. Social support affects state anxiety both directly and indirectly (through the mediation of trait anxiety). Therefore, during the COVID-19 pandemic, increasing public support for society can effectively reduce public anxiety.

## 1. Introduction

The COVID-19 pandemic, which is characterized by fast transmission and high infectivity, hit Wuhan, China, in December 2019 [1,2,3]. It spread quickly throughout China and beyond [4,5,6,7]. In the second half of January 2020, the first-level response to a public health emergency was established across China, and strong epidemic prevention measures were implemented. Some mandatory quarantine measures, such as the implementation of a travel ban [8,9], were adopted throughout China [10], and other policy measures were proposed successively, such as extending national holidays, closing schools, and postponing classes [11].

Public psychology is always affected by epidemics. Patients recovering from the SARS (severe acute respiratory syndrome) outbreak of 2003 had a higher than normal likelihood of emotional distress, including but not limited to anxiety, depression, and fear [12]. The 2012 Middle East respiratory syndrome (MERS) outbreak caused varying degrees of psychological trauma and severely affected quality of life [13,14,15]. The explosion of Ebola in West Africa in 2014 took a heavy toll on people and had a debilitating impact on public psychology [16,17]. Not only do people who experience traumatic events develop psychological symptoms, but also those who have been exposed to or associated with trauma experience varying degrees of psychological trauma, including post-traumatic stress disorder, melancholia, and depression [18,19,20]. Ample evidence has shown that psychological disorders (e.g., depression and anxiety) affect quality of life and physical and mental health [10,21,22]. Since the number of confirmed cases of COVID-19 have rapidly gone up and daily life and social activities have been restricted for an indeterminate period, people will inevitably suffer from anxiety and even become suicidal, potentially leading to longer-term damage to the mental health of individuals [14,23]. Thus, the psychological state of the public amidst the COVID-19 pandemic should be paid sufficient attention by decision-makers, researchers, and social media [24]. During such a crisis, the psychological health of society is arguably a major concern [25,26], and the implementation of mental health evaluation and support are priority goals for dealing with mental health consequences of COVID-19 [24].

Anxiety can be divided into state anxiety and trait anxiety [27]. State anxiety is a short-term emotional state generated by the perception of threatening stimuli, and it is used to assess people’s fear, tension, anxiety, and neurotic experience in immediate or recent occurrences. By contrast, trait anxiety refers to a relatively stable behavioral tendency of an individual to respond to various threatening stimuli, and it is often used to assess people’s personality traits [27].

Social support often means the care and support received from others [28]. It can reduce the anxiety level of people (e.g., patients) in stressful life events [29] and is positively correlated with mental and physical health [30,31]. Studies have revealed that social support can reduce depression and anxiety [32] and affect people’s psychological condition and alleviate anxiety, depression, and stress, contributing to post-disaster psychological recovery [21,33]. In order to improve public mental health in the context of the COVID-19 outbreak, the Chinese government has taken the following social support measures in terms of social support: (1) information support [34]—government and relevant departments are providing real-time disclosure of COVID-19-related knowledge and updates; (2) economic support—timely treatment is provided to confirmed patients, reducing financial stress and increasing public confidence; and (3) emotional support—the media reports on front-line health caregivers and their work to prevent and control the epidemic, uplifting public confidence in the fight against the virus. Moreover, most previous COVID-19 studies have focused on determining the epidemiological and clinical characteristics of infected patients [35,36], genomic characteristics of the virus [37], global health governance challenges [34,38], and mental health studies of front-line health care providers [39,40,41]. However, the existing literature on the psychological effects of COVID-19 on the general population in China is still scarce [8], and there are only a handful of studies devoted to this issue. Liu et al. [42] compared the differences in the mental state of the public among different genders, ages, occupations, education levels, and places of residence in the context of COVID-19, finding that gender and age have a significant impact on anxiety. Shevlin et al. [43] concluded that income is significantly related to the degree of anxiety caused by COVID-19.

In light of this, this study analyzes the public’s anxiety at the early stage of the COVID-19 outbreak in China, describes the anxiety characteristics of the respondents, compares the anxiety levels of multiple groups, and scrutinizes the relationship between social support and mental anxiety status of the respondents. This China-based study is of paramount importance for the accumulation of scientific evidence since China is where the virus was first officially reported. This study can provide support to governments and social institutions seeking to protect people’s mental health in mitigating the effects of the COVID-19 outbreak.

The remainder of this paper is structured as follows. Section 2 presents materials and methods. Section 3 shows and discusses the empirical results. Section 4 presents a discussion. Section 5 concludes the paper, summarizes research limitations, and lists avenues for future research.

## 2. Materials and Methods

### 2.1. Data Collection

This study was jointly conducted by researchers from several universities primarily located in Southwest China after Wuhan was closed down during the early COVID-19 outbreak. To explore the psychological health of the Chinese public in the context of the epidemic and the impact of social support on the mental state of the public, the authors initially designed an online structured questionnaire after examining existing literature and psychological evaluation scale manuals. It is worth noting that the online questionnaire survey is the only method available for the collection of anxiety and social support data at the early stage of the pandemic in China. Notably, the State–Trait Anxiety Inventory (STAI) is used to depict the anxiety profile of the public, and the Social Support Rating Scale (SSRS) is used to obtain the social support profile of the public.

The questionnaire was first filled out by all the team members to check whether or not the content is clear and unambiguous enough. Many university professors were then asked to check the validity of the questionnaire and point out minor problems to make the language of the questionnaire acceptable to the public. A pilot survey was then conducted by the team members’ relatives and friends between January 31st and February 9th, 2020.

The main survey was distributed through the Internet on February 10th. The questionnaire was distributed to various WeChat (a ubiquitous instant messaging/social media mobile phone application in China) groups. The respondents covered a wide range of places in China, including 33 provincial-level administrative regions. By the end of February 18th, 2020, a total of 956 questionnaires were collected, 736 of which were valid.

To facilitate the detection of a valid questionnaire and ensure the cleanness of data for further statistical analyses (data screening), we set 2 test questions in the survey: “This is a test question. Please select ‘3’ to show that you have read the question carefully” and “This is a test question. Please choose ‘2’ to show that you have read the question carefully”. If one or both of the questions were erroneous, the questionnaire was considered invalid. 

### 2.2. Ethical Context

The ethical contexts of this study are as follows. First, potential participants were well informed of the purpose of the research at the beginning of the questionnaire, and we promised that the data would be used only for scientific research and policy recommendations. After obtaining consent from respondents to participate in the research, a new page for questionnaire items would automatically open. Second, to preserve the anonymity of the respondents and ensure the confidentiality of the participant information, we decided not to include any personal identification items in the questionnaire.

### 2.3. Participants

Table 1 shows the socio-demographic characteristics of the participants. There were 429 (58.3%) females and 307 (41.7%) males. The respondents were mainly aged 18−25 years (30.3%), 26−30 years (19.2%), and 31−40 years (30.3%). 

### 2.4. Measure

#### 2.4.1. STAI-C

In this study, the STAI [27], which has been extensively used in research and clinical settings diagnosing anxiety and differentiating it from depressive syndromes, was used to measure the state anxiety and trait anxiety of the public. The STAI has recently been used in COVID-19 settings. A study used the STAI to investigate the state anxiety level of nurses offering care for COVID-19 patients in Turkey [44]. Another study adopted it to explore differences in anxiety among adults in the United Kingdom in the context of COVID-19 [45].

This study used the STAI with the COVID-19 background (STAI-COVID-19, hereinafter referred to as STAI-C) to depict the anxiety profile of the public. All the questions in the STAI-C were attached to the background of the epidemic, such as “before the occurrence of COVID-19, I felt happy” and “during the occurrence of COVID-19, I felt calm”. The STAI-C consists of the state anxiety inventory (SAI-C) and the trait anxiety inventory (TAI-C).

The STAI-C has 40 items, 20 for evaluating state anxiety and 20 for assessing trait anxiety. For state anxiety, 10 items describe negative emotions, and the other 10 capture positive emotions. For trait anxiety, 11 items reflect negative emotions, and the other 9 characterize positive emotions. 

The STAI-C uses a four-point scoring method. For the SAI-C, 1 = not at all, 2 = a little, 3 = somewhat, and 4 = very much so. For the TAI-C, 1 = hardly, 2 = some, 3 = often, 4 = almost always. All positive emotions are reversely scored.

The score of either state anxiety or trait anxiety ranges from 20 to 80. According to the score, people can be divided into 4 groups: no anxiety (≤20), mild anxiety (21–39), moderate anxiety (40–59), and severe anxiety (60−80) [22].

#### 2.4.2. SSRS

Social support is often defined as spiritual and material support from social networks that makes people feel cared for, loved, respected, and valued, and it positively impacts the mental health of disaster victims [31]. It is generally believed that social support can be divided into 2 categories in nature: one is objective, visible, or actual support, including direct material assistance and social networks, the existence of group relationships, and the degree of individual participation; the other is subjective and experienced emotional support, which refers to the emotional experience and satisfaction that an individual is respected, supported, and understood in society. In addition, the individual’s use of support should also be assessed. Individuals differ in their use of social support. Moreover, the support of people is an interactive process. While a person supports others, he/she may gain support from others.

This study adopted a 10-item SSRS designed by Xiao [46], which has been used in many prior studies [39,47,48]. The SSRS includes the following 3 dimensions: (1) subjective support dimensions (4 items), which reflect the subjective emotional experience and satisfaction of the respondents’ feeling; (2) objective support dimensions (3 items), which reflects the actual support, including direct assistance and social relations; and (3) support utilization dimensions (3 items), which reflect respondents’ utilization degree of social support. The higher the score, the higher the social support. In this study, no COVID-19-related information is included in the scale.

#### 2.4.3. Reliability and Validity Tests

In this study, Cronbach’s α and McDonald’s ω were adopted to test the reliability of the questionnaire data. The results showed that Cronbach’s α of the SAI-C was 0.919, Cronbach’s α of the TAI-C was 0.862, Cronbach’s α of the SSRS was 0.691, McDonald’s ω of the SAI-C was 0.920, McDonald’s ω of the TAI-C was 0.869, and McDonald’s ω of the SSRS was 0.734. Therefore, we can conclude that the reliability of the STAI-C data was at a high level, while the SSRS data had an acceptable level of reliability.

This study adopted a factor analysis to test the construct validity of the questionnaire data. The results revealed that the KMO (Kaiser–Meyer–Olkin) values of the state anxiety scale and trait anxiety scale were 0.933 and 0.921, respectively. Two factors were extracted from the SAI-C. The total variance explanation rate was 58.89%. Additionally, two factors were extracted from the TAI-C, and the total variance explanation rate was 50.71%. This result was compared with that of the original scale [27], and the entries in the two factors of state anxiety and trait anxiety in this study were consistent. The KMO of the SSRS was 0.713, and three factors were extracted. The total variance explanation rate was 55.16%. The items in the factor were consistent with the original scale [46]. Therefore, the questionnaire data had good construct validity.

### 2.5. Research Methods

In this study, the SPSS 24.0 statistical software was used for statistical analysis. Firstly, an exploratory analysis was then used to test the distribution and variance homogeneity of the scale data. If the data were normally distributed or homogenized, the *t*-test or analysis of variance (ANOVA) was used to check the score differences among different groups. Otherwise, the Mann–Whitney *U* test and the Kruskal–Wallis *H* test were used to check the score differences among different groups. Thirdly, a pair-wise correlation analysis was used to analyze the correlations among the SAI-C, the TAI-C, and the SSRS. Finally, the mediation effect was analyzed with a bootstrap set of 5000. *p* < 0.05 was considered “statistically significant” [49,50]. 

## 3. Results

### 3.1. Anxiety Levels of Different Socio-Demographic Groups

The average score of TAI-C was 38.0 ± 8.2, and that of SAI-C was 48.0 ± 10.4. The proportion of no anxiety symptoms was 0.5% (4 people), that of mild anxiety was 19.8% (146 people), that of moderate anxiety was 68.5% (504 people), and that of severe anxiety was 11.1% (82 people).

Table 2 reveals the mean and standard deviation of anxiety scores among socio-demographic groups. The average TAI-C score for males was 38.0 ± 7.7, and that for females was 38.0 ± 8.5. The average SAI-C score for males was 48.6 ± 10.6, and that for females was 47.5 ± 10.2. There were significant differences in state anxiety scores and positive emotion sub-score of state anxiety between family income groups ($\chi^{2}$ = 18.963, *p* < 0.01; $\chi^{2}$ = 22.295, *p* < 0.01). Except for the very poor and the very rich, the state anxiety level increased with advancing income. Another interesting observation was that the very rich had the lowest anxiety level. A possible explanation is that they have more resources and thus are more confident in combating the disease (e.g., they can pay more to find a cure). However, most characteristics seemingly did not affect anxiety levels. A possible explanation is that the state anxiety was already very high for all the respondents in the early stage of COVID-19. Future studies are needed to seek out more persuasive explanations.

### 3.2. Anxiety Differences among Social Support Groups

On the basis of the social support score, we divided the respondents into three groups: high, medium, and low. Respondents with a score less than or equal to 35 points were in the low group, and those with a score greater than or equal to 44 points were in the high group. Others (with a score between 35 and 44 points) were set as the medium group.

The results (Table 3) show that there were significant differences in the trait anxiety scale ($\chi^{2}$ = 64.989, *p* < 0.01). Similarly, state anxiety levels are significantly different among different groups.

### 3.3. Correlation Analysis between the STAI-C and the SSRS

Pearson correlation analysis was used in this study to further explore the correlation between various dimensions of state anxiety, trait anxiety, and social support. The analysis results are shown in Table 4. We observe that state anxiety and trait anxiety are positively correlated, while the social support measure is negatively related to the anxiety levels.

### 3.4. Relationship between Social Support and State Anxiety: Examining the Mediation Effect of Trait Anxiety

**Figure 1:**
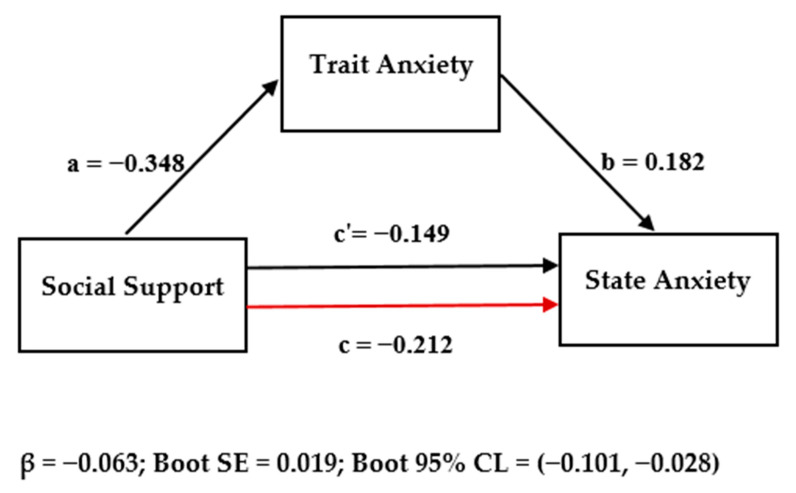
Mediation modeling results.

Trait anxiety may mediate the effect of social support on state anxiety. To examine such a mediation effect, we adopted the Hayes establishment of SPSS Model 4 (Model 4 for the simple mediation model) and used the bootstrap for several sets of 5000 [51]. The results (shown in Figure 1 and Table 5) revealed that social support had a significantly negative effect on state anxiety (*β* = −0.212, *p* < 0.01); the direct effect of social support on state anxiety was still significant when the mediating variables were added (*β* = −0.149, *p* < 0.01); and social support had a significantly negative effect on trait anxiety (*β* = −0.348, *p* < 0.01), while trait anxiety had a significantly positive effect on state anxiety (*β* = 0.182, *p* < 0.01). The direct effect (0.149) and mediation effect (0.063) of the model accounted for 70.24% and 29.76% of the total effect (0.212), respectively. 

## 4. Discussion

This study explores the anxiety of the Chinese public during the COVID-19 pandemic and the impact of social support on anxiety. It found that at the early stage of the pandemic, the respondents were generally anxious, with most presenting moderate anxiety. Our result is largely consistent with existing research results [8]. There may be several reasons for the high level of public anxiety: (1) COVID-19 seriously threatens the safety of life; (2) there is now no specific drug or vaccine against this virus [39]; (3) the first-level response adopted by the state was that all localities would be under closed management, and residents would not be allowed to enter or leave at will, thus restricting their mobility [52]; and (4) the excessive media exposure makes it hard to find a reliable information source, which makes people confused about what to do [53]. 

Therefore, the government should manage the highly credible information flow in an efficient, rapid, and transparent manner to reduce misinformation and speculation [52]. Once people get to know the latest development of the epidemic, they can follow the infection and recovery progress [8], better understand the merits of restricting outside movement and decrease interactions with others. Meanwhile, they can undertake recreational activities and physical exercise at home or in their neighborhood to maintain physical and mental fitness [9]. 

The sudden outbreak of COVID-19 has affected a wide range of people. The government has taken measures to quarantine them at home for some time. People who are isolated from their homes are unable to work (unless they work online), and family income may be severely affected; thus, there are inevitable concerns about the family’s economic functioning [11,52]. In addition, we find that gender, age, marital status, recent residential location, occupation, and education attainment do not affect trait anxiety or state anxiety. This result is different from other empirical results. A possible reason is that the life-threatening epidemic breaks out by leaps and bounds, and the anxiety of different groups of residents is all high. However, some socio-demographic characteristics (e.g., annual household income) truly affect people’s state anxiety [54,55,56,57]. This observation informs us that different people should be treated and addressed in different ways, and a uniform, one-size-fits-all method is not appropriate. 

People with different levels of social support are found to exhibit different levels of state anxiety and trait anxiety. Social support can effectively relieve anxiety, depression, and stress [39]. Friends or family members can provide social or emotional support, and social interactions can reduce negative emotions and improve mood [28,58]. As the number of confirmed COVID-19 cases in the country increases, more and more areas are affected, and residents are isolated at home and unable to go out to social events as usual, which aggravates the public’s anxiety [52,59]. If residents have extensive social networks, social support can reduce their psychological loneliness by reducing perceived and physical responses to the threat of stressful events as well as stress-induced inappropriate behavior [60].

The trait anxiety score and the state anxiety score showed a positive relationship. This result suggests that improving mental resilience, resilience to stress, and personality traits can make people more resistant to sudden irritant events [61,62]. Trait anxiety and state anxiety are negatively related to social support [11], which reveals that the higher the degree of social support, the lower the anxiety level. This finding indicates that social support is an effective intervention approach in people’s psychological conditions [39]. To reduce the public’s anxiety and promote psychological recovery of the public during/after the epidemic, the degree of social support for the public should be increased. For example, online and offline psychological counseling platforms can be set up. On the one hand, residents should have access to accurate information through broadcasting. On the other hand, psychological counseling can be provided to the public through electronic devices to actively and effectively ease the public’s anxiety [41,63].

The results of the mediation model show that social support has a direct effect as well as an indirect effect on state anxiety, and social support influences state anxiety by affecting trait anxiety. Individuals with higher levels of social support can better resist the negative effects of threat stimuli, and this habitual behavioral tendency is conducive to reducing the level of anxiety under the influence of COVID-19. Providing more social support at normal times is conducive to establishing a healthy and positive attitude and coping with unexpected events such as epidemics [11,64]. Therefore, effective psychological interventions and social support networks should be set up even in normal (non-crisis) periods to foster positive attitudes, improve personality traits and mental health, and enhance public resilience in the face of potential societal challenges [19]. Such efforts will also effectively help people recover faster in future calamity [65].

## 5. Conclusions

This study adopts the STAI and the SSRS to assess the levels of anxiety and social support in the early days of the COVID-19 epidemic. The results are as follows. First of all, in the early days of the COVID-19 pandemic, Chinese people are generally anxious since the pandemic seriously threatens physical health, with isolation restrictions on freedom adding to this anxiety. Second, anxiety level varies significantly among different social support groups. Third, social support is negatively correlated with the degree of anxiety. Put in another way, anxiety will be significantly lowered if social support is higher. More specifically, social support adversely affects state anxiety both directly and indirectly (through the mediation of trait anxiety). 

This study has some shortcomings. First, it is cross-sectional in nature. Future studies should use the longitudinal research method to compare the changes before and after the pandemic and analyze the influencing factors, which is more conducive to the construction of public psychological recovery after an epidemic. Second, one important dimension that has not been collected is financial resilience. It has been seen in crisis studies is that the level of savings, interfamilial ability and willingness to support, and access to infrastructure (e.g., ability to access funds) contributes to anxiety. Third, at the early stage of the epidemic, Chinese residents were isolated at home, so the only way to obtain the necessary information related to anxiety and social support for researchers was through an online questionnaire survey. In other words, researchers had no other methods available to collect the data during such a unique time. Admittedly, the online questionnaire data were not random (e.g., biased towards young undergraduates). This is a common weakness of early COVID-19 social science studies [42,45]. We agree that the mental health of the Chinese population should be assessed over the long run, and thus more rigorous data collection techniques and more sophisticated research methods are needed to reach a more robust conclusion.

## Figures and Tables

**Table 1:** Socio-demographic characteristics of the participants.

Characteristic	*N*	%	Characteristic	*N*	%
*Gender*			*Education attainment*		
Male	307	41.7	Junior secondary and below	30	4.1
Female	429	58.3	High school/technical secondary school/vocational high school	39	5.3
*Age*			Junior college	104	14.1
Below 18 years	1	0.1	Undergraduate	299	40.6
18−25 years	223	30.3	Master’s degree	170	23.1
26−30 years	141	19.2	Doctorate	94	12.8
31−40 years	223	30.3	*Annual household income (RMB)*		
41−50 years	113	15.4	Less than 30,000 (<USD 4560)	61	8.3
51−60 years	31	4.2	30,000−50,000 (=USD 4560−7600)	92	12.5
Above 60 years	4	0.5	60,000−100,000 (=USD 9120−15,200)	164	22.3
*Marital status*			110,000−150,000 (=USD 16,720−22,800)	122	16.6
Unmarried	315	42.8	160,000−200,000 (=USD 24,320−30,400)	100	13.6
Married	406	55.2	210,000−250,000 (=USD 31,920−38,000)	59	8
Divorced	12	1.6	260,000−300,000 (=USD 39,520−45,600)	52	7.1
Widowed	3	0.4	Over 300,000 (>USD 45,600)	86	11.7
*Occupation*			*Residential location*		
Ordinary employee/enterprise employee	238	32.3	Rural	122	16.6
Government/public institution personnel	215	29.2	Town	136	18.5
Farmer	9	1.2	City	478	64.9
Student	190	25.8			
Other	84	11.4			

**Table 2:** Anxiety levels of different socio-demographic groups ($\bar{x}$ ± *s*).

Characteristic	Category	Positive Emotion of TAI-C	Negative Emotion of TAI-C	TAI-C	Positive Emotion of SAI-C	Negative Emotion of SAI-C	SAI-C
*Gender*	Male (*n* = 307)	21.2 ± 5.9	16.8 ± 3.6	38.0 ± 7.7	29.8 ± 6.1	18.8 ± 6.6	48.6 ± 10.6
	Female (*n* = 429)	20.9 ± 6.4	17.1 ± 4.2	38.0 ± 8.5	29.1 ± 6.4	18.5 ± 6.0	47.5 ± 10.2
	*z*	−1.056	−0.615	−0.219	−1.341	−0.252	−1.152
	*p*	0.291	0.538	0.826	0.180	0.801	0.249
*Age*	<18 years (*n* = 1)	19	17	36	35	19	54
	18−25 years (*n* = 223)	21.1 ± 6.6	16.7 ± 3.5	37.9 ± 8.1	28.6 ± 6.8	18.6 ± 6.7	47.3 ± 11.2
	26−30 years (*n* = 141)	21.2 ± 6.3	16.3 ± 3.7	37.5 ± 8.1	30.0 ± 6.3	18.9 ± 6.4	48.9 ± 10.5
	31−40 years (*n* = 223)	20.6 ± 5.7	17.2 ± 4.3	37.8 ± 8.1	29.7 ± 5.6	18.5 ± 6.0	48.2 ± 9.6
	41−50 years (*n* = 113)	21.3 ± 5.8	17.4 ± 4.4	38.7 ± 8.5	29.2 ± 6.2	18.4 ± 5.7	47.6 ± 9.6
	51−60 years (*n* = 31)	22.2 ± 7.2	17.1 ± 4.6	39.3 ± 9.1	30.5 ± 7.2	19.1 ± 5.9	49.6 ± 11.0
	>60 years (*n* = 4)	25.3 ± 6.2	17.3 ± 1.7	42.5 ± 7.0	32.5 ± 3.7	17.5 ± 8.8	50.0 ± 9.5
	*H*	3.571	7.349	3.338	7.739	1.838	5.848
	*p*	0.734	0.290	0.765	0.258	0.934	0.440
*Marital status*	Unmarried (*n* = 315)	21.2 ± 6.4	16.8 ± 4.0	38.1 ± 8.4	29.1 ± 6.7	18.9 ± 6.6	48.0 ± 11.1
	Married/divorced/widowed (*n* = 421)	20.9 ± 6.0	17.0 ± 4.0	38.0 ± 8.1	29.7 ± 6.0	18.3 ± 5.9	48.0 ± 9.8
	*z*	−0.521	−0.537	−0.325	−1.051	−0.863	−0.402
	*p*	0.603	0.591	0.745	0.293	0.388	0.688
*Residential location*	Rural (*n* = 122)	20.8 ± 5.7	16.5 ± 3.7	37.3 ± 7.4	28.9 ± 6.1	18.2 ± 5.7	47.1 ± 9.8
	Town (*n* = 136)	21.4 ± 7.0	17.0 ± 4.4	38.3 ± 9.1	29.7 ± 6.7	18.8 ± 6.5	48.5 ± 10.9
	City (*n* = 478)	21.1 ± 6.1	17.0 ± 3.9	38.1 ± 8.1	29.5 ± 6.2	18.7 ± 6.3	48.1 ± 10.4
	*H*	0.788	2.493	1.084	1.541	0.529	1.622
	*p*	0.674	0.288	0.581	0.463	0.767	0.444
*Education attainment*	Junior secondary and below (*n* = 30)	19.6 ± 6.4	16.6 ± 4.1	36.2 ± 8.6	29.5 ± 6.3	18.3 ± 6.1	47.8 ± 10.0
	High school (*n* = 39)	21.2 ± 5.4	17.2 ± 4.5	38.5 ± 7.5	30.9 ± 4.9	18.6 ± 6.7	49.4 ± 10.5
	Junior college (*n* = 104)	21.0 ± 6.0	16.3 ± 3.0	37.2 ± 7.2	29.8 ± 6.4	19.4 ± 7.1	49.2 ± 11.3
	Undergraduate (*n* = 299)	21.1 ± 6.2	17.1 ± 4.1	38.2 ± 8.3	29.5 ± 6.4	18.4 ± 6.4	47.9 ± 10.6
	Master’s degree (*n* = 170)	21.3 ± 6.1	17.1 ± 4.2	38.4 ± 8.3	28.6 ± 6.4	18.9 ± 5.8	47.4 ± 10.0
	Doctorate (*n* = 94)	21.0 ± 6.9	16.9 ± 4.0	37.9 ± 9.0	29.7 ± 5.9	18.0 ± 5.3	47.7 ± 9.2
	*H*	2.442	2.497	2.706	4.991	2.634	1.817
	*p*	0.785	0.777	0.745	0.417	0.756	0.874
*Annual household income (RMB)*	Under 30,000 (USD 4560) (*n* = 61)	21.3 ± 6.6	16.8 ± 3.3	38.0 ± 8.1	29.7 ± 6.3	19.0 ± 6.8	48.7 ± 11.2
	30,000−50,000 (USD 4560−7600) (*n* = 92)	21.0 ± 5.8	17.3 ± 4.7	38.4 ± 8.7	29.1 ± 6.4	19.0 ± 6.2	48.1 ± 10.4
	60,000−100,000 (USD 9120−15,200) (*n* = 164)	20.0 ± 6.4	16.7 ± 4.1	36.7 ± 8.5	29.2 ± 6.6	18.9 ± 6.7	48.1 ± 10.9
	110,000−150,000 (USD 16,720−22,800) (*n* = 122)	21.5 ± 6.0	16.9 ± 4.2	38.4 ± 8.0	29.4 ± 6.0	18.7 ± 6.7	48.1 ± 10.5
	160,000−200,000 (USD 24,320−30,400) (*n* = 100)	22.1 ± 6.0	16.4 ± 2.9	38.5 ± 7.2	30.8 ± 5.7	18.3 ± 5.9	49.1 ± 9.9
	210,000−250,000 (USD 31,920−38,000) (*n* = 59)	21.9 ± 7.2	16.5 ± 3.9	38.5 ± 8.8	30.1 ± 6.0	18.5 ± 5.1	48.7 ± 8.0
	260,000−300,000 (USD 39,520−45,600) (*n* = 52)	20.8 ± 6.5	17.4 ± 4.3	38.2 ± 8.7	31.1 ± 6.1	19.6 ± 6.6	50.8 ± 10.6
	Over 300,000 (USD 45,600) (*n* = 86)	20.8 ± 5.1	17.7 ± 4.1	38.4 ± 7.6	27.0 ± 6.4	16.9 ± 5.1	43.9 ± 9.5
	*H*	10.271	7.071	8.353	22.295	8.071	18.963
	*p*	0.174	0.422	0.303	0.002	0.326	0.008
*Occupation*	Ordinary employee/enterprise employee (*n* = 238)	20.7 ± 6.0	16.6 ± 3.8	37.3 ± 7.7	29.9 ± 5.8	18.4 ± 6.4	48.2 ± 10.2
	Government/public institution personnel (*n* = 215)	21.7 ± 6.3	17.2 ± 4.2	38.9 ± 8.5	29.6 ± 6.2	18.3 ± 5.4	47.9 ± 9.4
	Farmer (*n* = 9)	19.2 ± 6.3	16.9 ± 3.5	36.1 ± 9.0	31.4 ± 5.5	20.6 ± 7.7	52.0 ± 12.0
	Student (*n* = 190)	21.4 ± 6.8	17.0 ± 4.1	38.4 ± 8.7	28.3 ± 7.2	18.8 ± 6.9	47.1 ± 11.8
	Others (*n* = 84)	19.8 ± 4.9	17.1 ± 3.6	37.0 ± 7.1	30.2 ± 5.6	19.3 ± 6.1	49.5 ± 9.5
	*H*	6.276	4.414	5.877	6.678	3.003	4.998
	*p*	0.179	0.353	0.209	0.154	0.557	0.287

**Table 3:** Comparison of anxiety levels between Social Support Rating Scale (SSRS) groups.

Item	SSRS Groups			$\chi^{2}$	*p*
	Low Social Support Group	Medium Social Support Group	High Social Support Group		
Number of observations	201	308	227		
Percentage (%)	27.3	41.8	30.9		
Positive emotion of TAI-C	22.8 ± 5.8	21.5 ± 6.1	19.0 ± 6.1	48.500	<0.01
Negative emotion of TAI-C	18.2 ± 4.4	17.0 ± 3.9	15.7 ± 3.2	51.258	<0.01
TAI-C	41.0 ± 8.2	38.5 ± 7.8	34.7 ± 7.5	64.989	<0.01
Positive emotion of SAI-C	30.3 ± 6.0	29.9 ± 5.9	28.1 ± 6.8	13.807	<0.01
Negative emotion of SAI-C	19.2 ± 6.5	19.0 ± 6.1	17.5 ± 6.1	11.976	<0.01
SAI-C	49.5 ± 10.1	48.8 ± 9.9	45.6 ± 10.8	16.762	<0.01

**Table 4:** Correlation analysis among scales.

	PE (TAI-C)	NE (TAI-C)	TAI-C	PE (SAI-C)	NE (SAI-C)	SAI-C	SS	OS	SU	SSRS
PE (TAI-C)	1									
NE (TAI-C)	0.258 **	1								
TAI-C	0.883 **	0.682 **	1							
PE (SAI-C)	0.227 **	0.059	0.201 **	1						
NE (SAI-C)	−0.077 *	0.309 **	0.092 *	0.366 **	1					
SAI-C	0.092 *	0.222**	0.178 **	0.828 **	0.825 **	1				
SS	−0.184 **	−0.269 **	−0.270**	−0.127 **	−0.098 **	−0.136 **	1			
OS	−0.200 **	−0.143 **	−0.221 **	−0.119 **	−0.072	−0.116 **	0.380 **	1		
SU	−0.196 **	−0.192**	−0.242 **	−0.07	−0.071	−0.085 *	0.387 **	0.312 **	1	
SSRS	−0.250 **	−0.264 **	−0.318 **	−0.146 **	−0.107 **	−0.153 **	0.819 **	0.797 **	0.620 **	1

Note: ** Significant at the 1% level. * Significant at the 5% level. (Positive emotion of TAI-C: PE (TAI-C). Negative emotion of TAI-C: NE (TAI-C). Positive emotion of SAI-C: PE (SAI-C). Negative emotion of SAI-C: NE (SAI-C). SS: subjective support; OS: objective support; SU: support utilization.).

**Table 5:** Mediation effect test of trait anxiety (*n* = 736).

Effects	Path	*β*	SE	*p*	
Effect: Social Support → Trait Anxiety	a	−0.348	0.038	<0.01	
Effect: Trait Anxiety → State Anxiety	b	0.182	0.048	<0.01	
Direct effect: Social Support→ State Anxiety	c’	−0.149	0.053	<0.01	
Total effect: Social Support→ State Anxiety	c	−0.212	0.051	<0.01	
State Anxiety total effect model (*F* = 17.637; *p* < 0.01; *R^2^* = 0.024)					
Indirect Effects		***β***	Boot SE	Boot 95% CI	
				IL	LL
Total indirect effect		−0.063	0.019	−0.101	−0.028

Note: Boot standard error, Boot CI lower limit, and Boot CI upper limit refer to the standard error, lower limit, and upper limit, respectively, of the 95% confidence interval of indirect effects estimated by percentile bootstrap method through deviation correction.
